# The structure, function, and distribution of gap junctions in the retina: Life cycle in health and disease

**DOI:** 10.1002/ccs3.70036

**Published:** 2025-09-03

**Authors:** Anna Pacwa, Klaudia Mroz, Xiaonan Liu, Adrian Smedowski

**Affiliations:** ^1^ Department of Opthalmology Faculty of Medical Sciences in Katowice The Laboratory for Translational Research in Ophthalmology Medical University of Silesia Katowice Poland; ^2^ GlaucoTech Co Katowice Poland; ^3^ Department of Physiology Faculty of Medical Sciences in Katowice Medical University of Silesia Katowice Poland; ^4^ Department of Ophthalmology Faculty of Medical Sciences in Katowice Professor K. Gibinski University Clinical Center Medical University of Silesia Katowice Poland; ^5^ Department of Ophthalmology Faculty of Medical Sciences in Katowice Medical University of Silesia Katowice Poland; ^6^ Institute of Biotechnology HiLIFE University of Helsinki Helsinki Finland; ^7^ Department of Paedriatric Ophthalmology Faculty of Medical Sciences in Katowice Medical University of Silesia Katowice Poland

**Keywords:** connexins, electrical synapses, gap junctions, intercellular communication, neurodegenerative diseases

## Abstract

Gap junctions are essential channels of communication between cells including neurons in the central nervous system. These channels coordinate cell metabolic and electrical functions including such crucial ones for maintaining homeostasis as cell proliferation, differentiation, survival, and apoptosis. They create narrow passageways that allow rapid exchange of small molecules, ions, and secondary messengers between neighboring cells including the retina and optic nerve. Disruption in normal functioning of gap junctions may result in various neurodegenerative disorders. A comprehensive understanding of gap junction composition, function, and regulation is the key in determining novel approaches to neuroprotection and neuroregeneration. Here, we review the structure and the role of gap junctions in the retina and discuss the life cycle of connexins and their involvement in retinal neurodegenerations.

## Graphical abstract

Gap junctions in healthy and diseases retina. Interneuronal gap junctions structured of connexin 36 and 45 present between retinal neurons provide passage for small molecules. Impaired conductivity of gap junctions participate in various retinal pathologies.

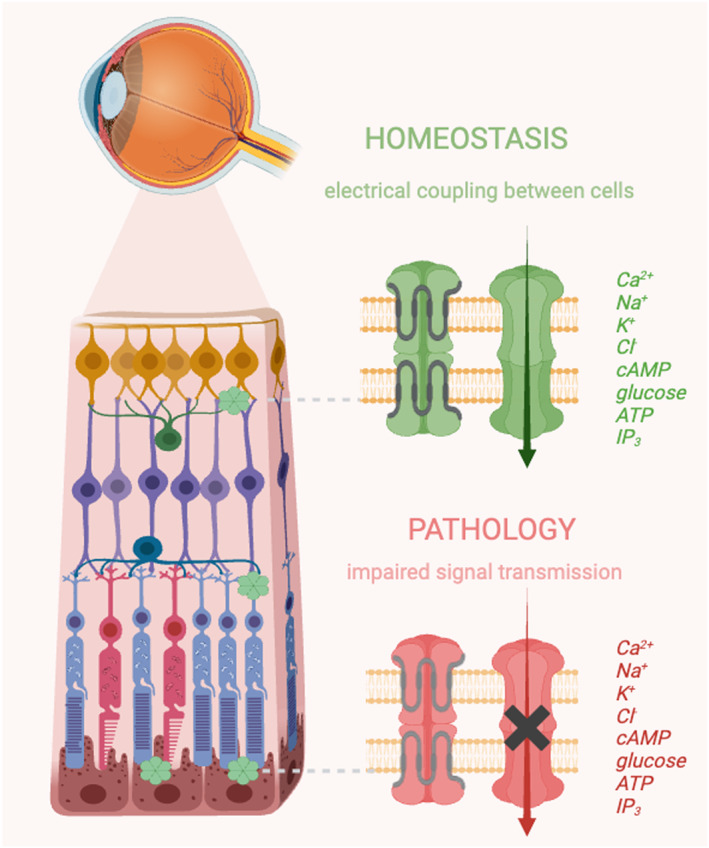

## 1 INTRODUCTION

Vision is the sense that people fear losing the most.^1^ The retina is the site of visual signal detection and initial processing. Retinal ganglion cells (RGCs) and their axons form the optic nerve, which is the only output pathway transmitting visual information to the primary visual centers in the brain^2^. Rapid inter‐neuronal communication is essential for the processing and propagation of signals within the brain. Understanding the fundamental mechanisms of signal transduction in neuronal cells is crucial for elucidating the pathological processes underlying vision loss.

There are two major communication pathways in CNS, direct and indirect, which play a crucial role in signal transmission. The mode of indirect transmission involves chemical synapses where neurotransmitters are released into the extracellular space and subsequently bind to receptors on the postsynaptic cell membrane.^2^, ^3^ These receptors are highly selective to their corresponding transmitter which regulates the excitation, inhibition, or modulation of the post‐synaptic neuron. The chemical synapses systems are very sophisticated with high energy demand.^4^, ^5^, ^6^ In contrast, direct communication mediated by electrical synapses allow for passive transport of ions and molecules, independently from energy input.^7^, ^8^ Electrical synapses conductivity depends on the channel properties and the signal passes without synaptic delay which is typical for chemical synapses. All these features make electrical synapses a rapid and precise system for action potential propagation and modulation, particularly in syncytial arrangement of excitable cells (e.g., neurons or muscles).^9^, ^10^ A remarkable example of this type of communication is found in the vertebrate retina, where five major neuron types are interconnected through gap junctions (GJs) expressing various connexin (Cx) proteins.^5^, ^6^ Both neuronal cells, including RGC, amacrine cells, bipolar cells, horizontal cells, and photoreceptors, and non‐neuronal cell, such as astrocytes, microglia, and Müller cells, commonly form GJs, which facilitate electrical and metabolic communication, playing a crucial role in both signal transmission and cellular metabolism under physiological and pathological conditions. Thus, the retina represents one of the most optimal model systems for investigating the functional roles of neuronal gap junctions and electrical transmission in the CNS.

## 2 STRUCTURE AND FUNCTION OF GAP JUNCTIONS

Electrical synapses (gap junctions) have received considerable interest in recent years as they constitute a key element of the structure and function of the CNS allowing fast and simple intercellular communication. Gap junctions were first reported almost 70 years ago^4^, ^8^ and as important components of retinal circuitry GJs were identified over 50 years ago.^7^

By definition, gap junctions are specialized connections that ensure communication between neighboring cells, including neurons, which allow the rapid transfer of nutrients, metabolites, and ions directly between cells, bypassing extracellular space.^10^ Gap junctions, serving as the structural basis for electrical synapses, consist of two hemichannels, one inserted into each apposing membrane, known as connexons. These connexons bridge an extracellular gap of 2–4 nm, forming channels that directly connect the cytoplasm of adjacent cells. This connection facilitates the intercellular diffusion of molecules up to approximately 1 kDa. Each channel is assembled from transmembrane protein subunits known as connexins. Twelve connexins assemble to create a functional gap junction channel; six connexins in one cell's plasma membrane oligomerize into a hemichannel, which then aligns and connects with a corresponding hexamer on a neighboring cell.^11^ Connexins are generally defined according to their molecular weights between 21 and 70 kDa and described by the formula CxMW (e.g., Cx43, Cx35, Cx40, etc.).^9^ Connexins are synthesized in the endoplasmic reticulum, where they assemble into hexameric connexons, either within the endoplasmic reticulum or the endoplasmic‐Golgi intermediate compartment. Subsequently, they are transported to the Golgi apparatus and delivered to the cell membrane through vesicular or direct transport mechanisms, relying on a microtubule‐dependent pathway.^12^

**FIGURE 1:**
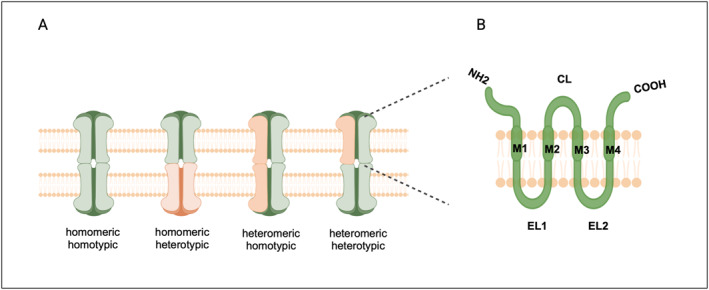
The composition of gap junctions. The oligomerization of six connexins forms a hexamer named connexons or hemichannel (A). Hemichannels can be homomeric, consisting of a single connexin isoform, or heteromeric, containing more than one connexin isoforms. Similarly, gap‐junction channels can be homotypic, formed by identical hemichannels, or heterotypic, composed of different hemichannels. (B) Basic structure of connexin protein. M1‐M4‐ transmembrane domains, EL1, El2‐extracellular loop domains, CL‐cytoplasmic loop domain, NH2‐ amino‐terminal end, and COOH‐ carboxy‐terminal ends.

Connexons can be homomeric, composed entirely of one connexin type (e.g., Cx36/Cx36), or heteromeric, comprising different connexin types (e.g., Cx43/Cx32). However, functional hemichannels typically form only from specific connexin combinations. Furthermore, intercellular channels can be composed of either homotypic connexons where both hemichannels are identical, or heterotypic connexons which involve the alignment of different connexon types^13^ (Figure 1A). Structurally, connexins have four transmembrane domains (M1‐M4), two extracellular loop domains (EL1, EL2), cytoplasmic amino‐terminal and carboxy‐terminal ends (NT and CT), and a single cytoplasmic loop domain (CL).^14^ More than 20 connexin isoforms sharing a similar topology have been identified, with variations primarily occurring in the lengths and sequences of their cytoplasmic loops and carboxyl‐terminal domains^15^ (Figure 1B). Various cells may express different connexin subunits to form gap junctions depending on the function served. Gap junctions allow the passage of small ions (Ca^2+^, K^+^, Na^+^, and Cl^−^), signaling molecules (ATP, cAMP, and IP3), nutrients (glucose and amino acids), neurotransmitters (glutamate), and larger linear molecules such as polypeptides and siRNA between connected cells.^16^, ^17^ In humans and mice, about 20 different connexin isoforms have been identified,^6^ each connexin exhibits distinct properties and is expressed in specific type of tissue.

The most highly expressed connexin in human cells and thereby the most widely described connexin is Cx43.^12^, ^18^ The expression levels of connexins may vary between tissue types. In human retina, for instance, the highest expression levels are detected for connexins 36, 43 and 45.^2^, ^9^, ^19^, ^20^, ^21^ There are gap junctions found in meningothelial cells (composed of Cx36)^22^ and GFAP‐positive optic nerve astrocytes (composed of Cx43).^23^, ^24^, ^25^, ^26^ In the optic nerve (ON), previous studies have shown that GJ formed by Cx43 establish connections between astrocytes and neighboring axons.^27^ However, axons themselves function as isolated pathways, conducting action potentials directly toward the brain without collateral impulse spreading. This structural organization ensures precise signal transmission but also makes the conduction process highly vulnerable. If an axon becomes damaged or experiences a disruption, the lack of alternative pathways means that action potential conduction could be blocked, potentially leading to functional impairments in visual processing.

Smedowski et al.^28^ has recently discovered that in the optic nerve head gap junctions, comprised predominantly of Cx45 and Cx36, are present between axons. The identification of these connexins suggests that electrical synapses play a significant role in synchronizing RGCs activity, enhancing the efficiency of neural signaling and reducing signal loss. Facilitating crosswise conduction within bundles of the ON may provide an alternative pathway for signals, allowing them to bypass localized damage in axons. These findings provide new insights into optic nerve physiology and open up potential avenues for therapeutic strategies aimed at preserving visual function in diseases affecting the optic nerve.

## 3 VISUAL PROCESSING AND GAP JUNCTIONS

Neuronal cell–cell communication by gap junctions is essential for visual processing. In the retina, there are five classes of neurons, including photoreceptors, horizontal cells, bipolar cells, amacrine cells, and ganglion cells.^29^ Each class has been associated with gap junction coupling and thereby modulating light signal transduction pathway. Connexins expression in the retina varies by cell type (Table 1 summarizes the different connexins in human retina). This diverse distribution of connexins indicates that gap junctions play a significant role in transmission of light signals. Factors such as subunit composition, post‐translational modifications, as well as the number and placement of gap junction channels, contribute to the diverse functions of these structures in maintaining cellular homeostasis and regulating signal transmission.^34^

**TABLE 1:** Expression pattern of connexins in retina.

Cell type	Connexin type	References
Photoreceptors	Cx36	30
Horizonal cells	Cx50, Cx57, Cx62	31
Bipolar cells	Cx36, Cx45	32, 33
AII amacrine cells	Cx36, Cx45	34, 35
Retinal ganglion cells	Cx36, Cx45, Cx30.2	36, 37, 38
Muller cells	Cx30, Cx30.3, Cx32, Cx43, Cx45, Cx46	39, 40
Astrocytes	Cx43, Cx30, Cx26, Cx45	41, 42
Microglia	Cx32, Cx36, Cx43	43, 44, 45, 46, 47, 48, 49, 50, 51

**FIGURE 2:**
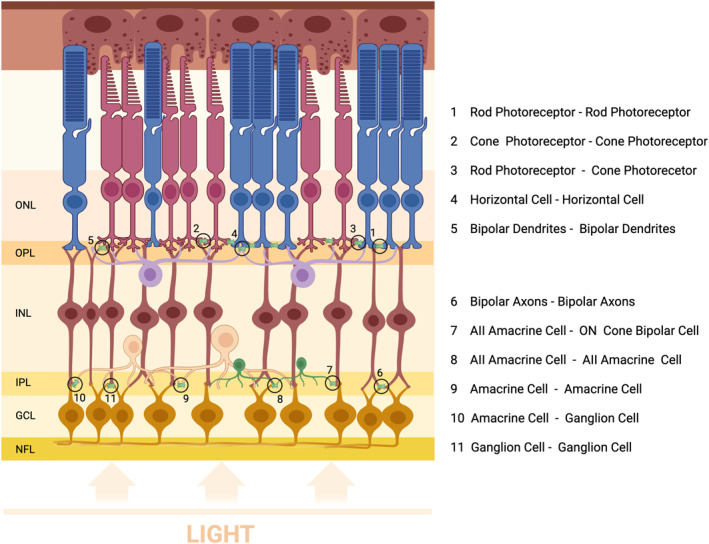
Schematic representation of gap junctions in the retina. The retina is shown in vertical cross‐section, including photoreceptors (rods and cones), horizontal cells, bipolar cells, amacrine cells (notably AII amacrine cells), and ganglion cells. Gap junctions, primarily composed of connexin, are represented as green hexagons. NFL‐ Nerve Fiber Layer, GCL‐ Ganglion Cell Layer, IPL‐ Inner Plexiform Layer, INL‐ Inner Nuclear Layer, OPL‐ Outer Plexiform Layer, and ONL‐ Outer Nuclear Layer.

Retinal neurons are arranged into cellular layers, including the outer nuclear layer (ONL), the inner nuclear layer (INL), and the ganglion cell layer (GCL). There are also inner and outer plexiform layers (IPL and OPL) that are involved in visual processing by facilitating synaptic contact among all types of neurons (Figure 2). The direct pathway for processing light information from photoreceptors to the optic nerve is a three‐neuron chain‐photoreceptor cell to bipolar cell and next to the retinal ganglion cell.^52^

Photoreceptors detect light, with rods enabling vision in low‐light conditions and cones responsible for color perception and high‐acuity vision in bright light. Photoreceptors have an outer segment (containing visual pigment molecules) that is partly embedded in the pigment epithelial layer and the inner segment, rich in mitochondria and ribosomes. The cell body, which contain the nucleus, is located in the ONL, whereas the synaptic terminals reside in the OPL where they form synapses with both bipolar and horizontal cells.^9^ Pigment molecules absorb light and this results in changes in the membrane potential of rods and cones. This alters the rate at which neurotransmitter is released by photoreceptor cells at their synapses with the bipolar cells, whose cell bodies reside in the INL. Neurotransmitter released from photoreceptors modulates the membrane potential of bipolar cells, thereby influencing the rate at which they release neurotransmitters into retinal ganglion cells. This, in turn, regulates the frequency of action potential generated in RGC. Bipolar cells establish synaptic connections with RGC within the inner plexiform layer (IPL). The axons of RGC form the optic nerve which transmits visual information to the brain.^52^

The remaining two classes of retinal neurons, horizontal cells and amacrine cells with cell bodies localized in INL, mediate lateral communication within the retina. The main function of horizontal cells is to modulate the light sensitivity, sharpening the perception of contrast between light and dark conditions. Amacrine cells are responsible for the formation of local interconnections between bipolar cells and ganglion cells.^52^ Their roles, unfortunately, are not yet fully elucidated. There are several different amacrine cell types and each of them performs specific functions in visual pathway. For example, one cell type seems to be crucial during the transmission of visual signal from rod photoreceptors to ganglion cells. In general, horizontal and amacrine cells are responsible for the inhibition or facilitation (feed‐back or feed‐forward) of communication between photoreceptors and ganglion cells thus modulating retinal sensitivity.^9^ The GCL contains ganglion cells and some displaced amacrine cells. Finally, the axonal fibers of ganglion cells gather in the thin nerve fiber layer (NFL) and leave the retina through the optic disc.

In the majority of vertebrate species, photoreceptors are interconnected through electrical synapses, including homologous connections between rods or cones and heterologous connections between rods and cones. One important function attributed to gap junctions in rod‐cone coupling is their ability to provide an alternative signaling pathway through cones for processing light detected by rods under scotopic conditions.^53^, ^54^ Only a limited connexin types are known to form gap junctions in the photoreceptor synaptic terminals. Among them, Cx36 is specifically found in cone photoreceptors,^31^, ^33^ although some studies suggest that rod photoreceptors may also contain Cx36 gap junctions.^31^ Studies in Cx36 knockout mice show complete inhibition of rod‐mediated signaling at the ganglion cell layer.^55^ Surprisingly, other work found that in Cx36 knockout models, retinal development proceeded normally without any alterations in cellular organization. The study concluded that Cx36‐containing gap junctions are essential for proper synaptic transmission within the rod pathway. However, it could not confirm the presence of Cx36 in rod photoreceptors themselves.^56^ Therefore, it is possible that rod photoreceptors are either interconnected with the cone signaling pathway or express other connexin proteins.^57^

Next cell type which modulate the visual signal through gap junctions in the visual pathway are horizontal cells. Experimental research indicate that different subtypes of horizontal cells create only homologous gap junctions with their properties varying according to their location in the membrane of the different cellular compartments.^58^, ^59^ Gap junctions of horizontal cells in mice express Cx50 and Cx57. Specifically, some studies have shown that axonless horizontal cells in mammals express Cx50, whereas axon‐bearing horizontal cells express Cx57.^34^, ^60^ Cx57 is essential for horizontal cell receptive field size, as its knockout drastically reduces lateral signal integration.^61^ Its human homolog, Cx62, has been localized to the OPL, IPL, and GCL.^62^ In zebrafish, a closely related group of connexins (Cx52.6, Cx52.7, Cx52.9, and Cx55.5) expressed by horizontal cells, is believed to participate in their electrical coupling.^63^

Cone photoreceptors communicate with horizontal and bipolar cells through Ca^2+^‐dependent glutamate release. When exposed to light, cones hyperpolarize, decreasing glutamate release. Horizontal cells, which are electrically coupled and receive input from multiple cones, integrate these signals across a wide area. They then provide inhibitory feedback to cones, creating the center‐surround receptive fields of bipolar cells. This feedback occurs via an ephaptic mechanism, a unique type of neuronal signaling that modulates the extracellular electric potential.^64^

In mammals, bipolar cells vertically oriented glutamatergic interneurons relay signals from the outer to the inner retina. Some bipolar cells establish gap junctions with each other, but they also connect via gap junctions with other cell types, especially amacrine cells. Connection between bipolar cells happens within ON cells and within OFF cells, but not between ON and OFF cells. Gap junctions that mediate communication between bipolar cells consist of Cx36 which is found mainly in OFF bipolar cells.^65^ In mice, the expression of Cx45 was found in OFF and ON cone bipolar cells.^66^ Bipolar cells can also communicate with a subtype of amacrine cells, known as AII cells, which form gap junctions with ON cone bipolar cells.^67^ Whereas Cx36 is expressed in OFF bipolar cells, it is also present in AII amacrine cells.^65^ ON cone bipolar cells and AII amacrine cells are capable of forming heterotypic gap junctions that consist of Cx45 and Cx36, respectively. Genetic studies revealed that blocking the Cx45/Cx36 gap junction affects rod signal transmission.^56^ In humans, Cx45 and Cx36 have been detected in the inner and outer plexiform layers.^62^ The inhibition of Cx36 has similar effects on signal transduction pathway as deletion of Cx45.^56^ AII amacrine cells can communicate with each other via gap junction, which are primarily composed of the Cx36.^68^, ^69^ Different subtypes of amacrine cells such as the parvalbumin positive/glycinergic amacrine and A3 amacrine cells are capable of forming gap junction between themselves.^70^

Ganglion cells are the only projection neurons, meaning they are the sole carriers of information from the vertebrate retina to the visual centers of the brain. Gap junctions can occur both between ganglion cells and between ganglion cells and amacrine cells.^71^ Interestingly, no reports have documented coupling between different ganglion cell subtypes, suggesting that their gap junctions support distinct electrical networks within the inner retina. In rats and mice, some ganglion‐to‐ganglion cell connections are formed by Cx36 or Cx45 and Cx30.2.^72^, ^73^, ^74^ However, the identity of many homotypic ganglion cell gap junctions remains unclear. Additionally, the molecular composition of ganglion cell gap junctions in other vertebrate species is still largely unknown. RGCs axons form the optic nerve, where electrical synapses formed by neuronal connexins, particularly Cx45, establishing direct structural and functional connections between them.^28^

Although neurons play a central role in retinal signaling, glial cells are equally important for maintaining retinal integrity and function. Retinal glia consists predominantly of three cell types: Müller cells, astroglia, and microglia. Müller cells provide structural support, perform metabolic functions, and facilitate neural signal transmission. Studies have shown that subpopulations of isolated Müller cells express mRNA for several connexins including Cx30, Cx30.3, Cx32, Cx43, Cx45, and Cx46.^39^, ^40^ Astrocytes contribute to the maintenance of the blood‐retina barrier, supply essential nutrients, and regulate retinal blood flow.^75^ The predominant connexin isoform forming astrocytic gap junction channels is Cx43 with additional contributions from Cx30, Cx26, and Cx45.^41^, ^42^ Microglia, the resident immune cells of the retina, are responsible for clearing cellular debris, responding to inflammation, and protecting against pathogens.^43^ In the CNS, microglia express connexins such as Cx32, Cx36, and Cx43; however, in their resting state, they form only a limited number of functional gap junctions.^44^, ^45^, ^46^, ^47^, ^48^, ^49^ Activation of microglia upregulate Cx43 expression, although it appears to be absent in brain‐resident microglia.^50^, ^51^

## 4 LIFE CYCLE OF CONNEXINS

Connexin proteins are synthesized in the cytoplasm and the endoplasmic reticulum (ER). Connexins assemble into connexons within the ER or the ER‐Golgi intermediate compartment. Chaperones like ERp29 ensure proper connexin folding, preventing misfolding or aggregation during this critical phase.^76^ Within the ER or the ER‐Golgi intermediate compartment (ERGIC), six connexin subunits assemble into a hexameric structure known as a connexon (or hemichannel), a process tightly regulated by cellular quality‐control mechanisms.^11^ Connexons are subsequently trafficked to the Golgi apparatus, where they undergo post‐translational modifications such as phosphorylation which fine‐tune their stability, membrane localization, and channel gating properties.^77^ From the Golgi, connexons are transported to the plasma membrane through vesicular carriers or direct membrane fusion, where they dock with adjacent connexons.^17^ Docking relies on precise interactions between extracellular loop domains, with calcium ions stabilizing these intercellular connections to form functional gap junction channels. These channels aggregate into dense plaques containing hundreds to thousands of connexons, creating conduits for ions, metabolites, and signaling molecules.

The half‐life of gap junctions typically ranges from 1.5 to 3 h depending on the connexin isoform, necessitating rapid turnover to maintain dynamic cellular communication.^78^ It is generally accepted that during normal physiological conditions, connexons are regularly incorporated at the periphery of existing gap junctions, while those in the central region are internalized through endocytosis.^79^ However, connexons remain connected, and the gap junction is internalized by one of the cells.^80^ The internalized gap junction structure is often referred to as connexosome.^81^ Gap junction internalization involves endosomal degradation, where the plasma membrane of one cell attaches to the internalized plasma membrane, forming a double‐membrane structure. This is then directed for degradation by lysosomes and proteasomes.^81^

## 5 REGULATION OF GAP JUNCTIONS

Gap junctions are highly dynamic structures that can be regulated through various mechanisms. They react to intracellular signals by altering their conformation and closing their channels. This occurs, for instance, when a cell experiences damage. Consequently, essential metabolites may leak out, whereas ions like Ca^2+^ and Na^+^ flow into the cell.

During the early stages of neurodegeneration, cell coupling is inhibited, allowing healthy cells to remain unaffected due to the plasticity of gap junctions and the closure of their channels. Recent findings suggest that gap junction coupling is regulated by Ca^2+^ via calmodulin binding.^82^ When influenced by Ca^2+^, calmodulin binds to connexin, leading to a reduction in gap junction conductance or even complete closure of the junctions.^83^

The retina adapts to a vast range of light intensities through various mechanisms, including shifts in rod and cone photoreceptor pathways. A key adaptation involves Cx36 GJs between rods and cones which dynamically regulate signal transmission.^84^ In darkness, GJs open, allowing rod signals to reach cone pathways, while in bright light, they close because of dopamine‐mediated inhibition of Cx36 phosphorylation. Adenosine also modulates this process, enhancing coupling at night and suppressing it during the day.

Beyond photoreceptor GJs, light adaptation affects other retinal circuits. Horizontal cells adjust their coupling in response to ambient light via dopamine and nitric oxide signaling, influencing receptive field size.^85^ AII amacrine cells alter their coupling depending on light intensity, with Cx36 and Cx45 contributing to different conductance properties.^86^ Similarly, RGCs expand their coupling networks under light adaptation, modifying their response properties. Long‐term light exposure influences connexin expression, with Cx36, Cx43, Cx45, and Cx57 transcript levels decreasing in prolonged darkness.^87^ These findings suggest that natural light cycles regulate connexin expression and GJ function, contributing to retinal adaptation.

Intracellular pH plays a critical role in regulating gap junction coupling between cells. It is widely accepted that intracellular acidification typically leads to a reduction in gap junction conductance^88^, whereas intracellular alkalization enhances conductance.^35^ However, alkalosis has been observed to decrease the conductance of gap junctions composed of Cx36, while acidosis has been shown to increase their conductance.^89^

Post‐translational modifications may also contribute to the regulation of gap junctions. So far, several different mechanisms have been described in the literature: phosphorylation, ubiquitination, SUMOylation, acetylation, hydroxylation, nitrosylation, and methylation.^90^ Multiple protein kinases mediate the phosphorylation of connexins, a key post‐translational modification that plays a crucial role in various stages of the connexin life cycle. Phosphorylation is implicated in the regulation of connexin trafficking, assembly into gap junctions, disassembly, and modulation of gap junction conductance.^91^

The literature indicates that there is an interplay between phosphorylation and ubiquitination of connexins in tissue cells. The phosphorylation of connexins (Cxs) plays an important role in regulating gap junction (GJ) physiology. This post‐translational modification has been closely linked to several key aspects of GJ biology including the number of functional gap junctions present at the plasma membrane, the remodeling and dynamic reorganization of gap junction plaques, and the internalization and subsequent degradation or recycling of GJs.^92^, ^93^ Multiple types of kinases are involved in mediating these phosphorylation events with notable contributors including protein kinase A (PKA), protein kinase C (PKC), mitogen‐activated protein kinases (MAPKs), casein kinase 1 (CK1), and the non‐receptor tyrosine kinase Src. Each of these enzymes can target specific serine, threonine, or tyrosine residues on connexin proteins, thereby influencing their assembly, stability, and turnover within cellular membranes. Through these regulatory pathways, phosphorylation serves as a central mechanism for controlling intercellular communication via gap junctions in both physiological and pathological contexts.

In addition to phosphorylation, recent studies demonstrate that SUMOylation is also an important post translational modification of connexins.^94^ SUMOylation is defined as a reversible process involving post‐translational protein modifications.^95^ The stabilization of connexins is dependent on small ubiquitin‐like modifier (SUMO) protein necessary for SUMOylation. Enzymes that are required for SUMOylation and various SUMO isoforms tend to be tissue and cell specific. There is some evidence to suggest that SUMO2 is the most frequently expressed protein isoform in retina.^96^

**FIGURE 3:**
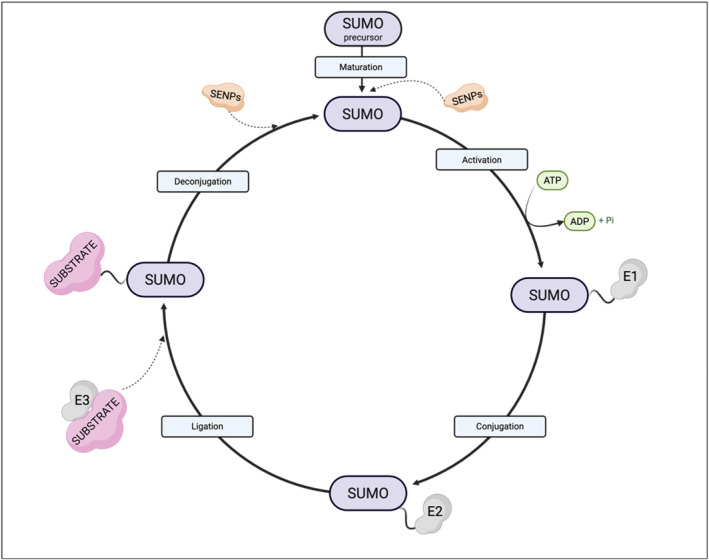
Mechanisms of SUMOylation. SENP enzymes convert the SUMO precursor into its mature form. SUMO is activated through an ATP‐dependent reaction carried out by the heterodimeric E1 enzyme. The activated SUMO is transferred to a cysteine residue on the E2 enzyme. Then is covalently attached to a specific lysine residue on the target protein. While the E2‐SUMO complex can mediate this step alone, it typically requires an E3 ligase to enhance the efficiency and specificity of the conjugation. SENP enzymes remove SUMO from substrate proteins, releasing free SUMO molecules.

SUMOylation is not yet fully understood because of its complexity. Recent documents reveal that three key groups of enzymes are required for the process of SUMOylation: E1 activating enzyme (known as SAE1/2 in retina), E2 conjugating enzyme (UBC9 in retina) and E3 ligase (CBX4, HDAC4, PIAS3, TLS, and TOPORS in retina).^96^ Additionally, there is a SENP enzyme which has two important functions. The first role as endopeptidase, is to remove C‐terminus chain and convert preSUMO into mature SUMO. The second role as isopeptidase is deSUMOylation and recovery of SUMO protein by disconnecting it from target protein^95^ (Figure 3).

Studies have shown a connection between SUMOylation and the life cycle of connexins, particularly Cx43. Kjenseth et al. demonstrated that the SUMO system directly influences Cx43 protein stability and membrane localization, thereby contributing to the regulation of gap junctional intercellular communication.^94^ Specifically, they identified two membrane‐proximal lysine residues—Lys144, located within the intracellular loop, and Lys237, situated in the C‐terminal tail—as primary SUMO conjugation sites on Cx43. Mutational analyses and biochemical assays confirm that these residues serve as functional SUMO acceptor sites essential for mediating the observed regulatory effects.

Collectively, these findings not only establish Cx43 as a target of SUMOylation but also provide the first direct evidence implicating the SUMO modification pathway in the post‐translational regulation of gap junction assembly and maintenance. These results expand the current understanding of the molecular mechanisms governing GJ homeostasis and highlight SUMOylation as a novel modulator of connexin function.^13^

## 6 THERAPEUTIC APPROACHES TO RETINAL DISEASE

Retinal neurons are vital components of the CNS and lack the ability to regenerate spontaneously after injury. Consequently, damage leads to the irreversible loss of RGCs.^97^, ^98^ GJs are increasingly recognized as crucial mediators of retinal neurodegeneration, particularly in diseases such as ischemic retinopathy, retinitis pigmentosa, and glaucoma.^99^ Numerous researchers studying neurodegenerative diseases have sought to identify the factors that contribute to their development. The phenomenon of cell‐cell coupling is under the investigation of several research groups. So far studies that have noted the importance of cell coupling indicate both beneficial as well as harmful effects of GJs formation.^100^, ^101^

GJs can provide neighboring cells with nutrients (e.g., glucose) as well as regulatory molecules (e.g., cyclic AMP, ADP, and glutathione), which suggests their positive involvement in cell survival processes. However, the bystander effect, in which dying cells transmit harmful metabolites to neighboring neurons via GJs, contributes substantially to disease progression.^102^ This can be detrimental, potentially triggering excitotoxicity, neurodegeneration, and the activation of intracellular cell death pathways. The concept of secondary neurodegeneration associated with gap junctions is discussed in literature.^103^ In summary, the primary insult that triggers neurodegeneration primarily impacts the most vulnerable cells. Studies indicate that RGCs undergoing apoptosis trigger degeneration in adjacent neurons coupled via GJs, leading to extensive cell loss in the inner retina.^104^, ^105^ More and more gap junctions are assembled what eventually leads to the spread of secondary neurodegeneration and consequent neuronal damage.

Blocking GJs under excitotoxic or ischemic conditions increases RGC survival by approximately 70%, demonstrating that intercellular coupling significantly contributes to neuronal loss.^19^ Additionally, genetic deletion of connexins Cx36 and Cx45 offers neuroprotection by disrupting toxic signaling pathways. Cx36 deletion significantly reduces RGC death by approximately 50% under excitotoxic conditions, whereas cell loss in Cx45 knockout mouse retinas was comparable to wild‐type mice. Conversely, ablation of Cx45 reduced neuronal loss by about 50% following ischemic insult, whereas deletion of Cx36 did not confer protection.^19^ Immunolabeling studies show that Cx36 and Cx45 exhibit distinct expression and trafficking patterns under pathological conditions. Cx36 levels are reduced after ischemia and remain unchanged after excitotoxicity, whereas Cx45 levels are reduced following excitotoxicity and remain unchanged after ischemic insult. This suggests that different types of GJs, depending on their connexin composition, contribute to cell death under distinct pathological conditions.

Connexin 43 is the most widely studied channel protein in the optic nerve and so any potential therapeutic tactics generally take this specific protein into consideration while designing treatment strategies.^106^, ^107^, ^108^ In the retina, particularly in the posterior eye, Cx43 plays an important role in supporting retinal homeostasis. However, following injury, Cx43‐mediated gap junctions and hemichannels can shift from being protective to pathological. After partial optic nerve damage, Cx43 is often upregulated in the retina.^109^ The increase is associated with an inflammatory response and degeneration of RGC.

One proposed mechanism involves the spread of toxic ions and metabolites from dying to healthy cells through Cx43 gap junctions.^110^ Additionally, the activation of unpaired Cx43 hemichannels may allow the release of signal molecules into the extracellular space, contributing to further tissue injury and cell death. Numerous studies indicate that the release of apoptotic or necrotic signals from injured cells into the extracellular space is propagated primarily through open hemichannels rather than directly via gap junctions.^111^ ^,^ ^112^

Mimetic peptides such as Gap26 and Gap27 are synthetic peptides designed to mimic short sequences of amino acids found on the extracellular loops of connexin proteins.^113^ They function by binding to these extracellular regions, which allows them to rapidly inhibit hemichannel activity—typically within minutes. Additionally, when applied over longer periods, these peptides prevent the docking of opposing hemichannels, thereby disrupting the formation of functional gap junctions and inhibiting intercellular communication.^114^ ^,^ ^115^ The Gap19 mimetic peptide selectively blocks hemichannel opening while preserving gap junction coupling, presenting new therapeutic opportunities.^114^ ^,^ ^116^ Using mimetic peptides to target Cx43 after ocular injury has shown potential to reduce retinal damage, inflammation, and neuronal loss.^117^ Furthermore, intravitreal injections of these peptides have been found to protect RGCs by decreasing vascular leakage and hemichannel activity after ischemic injury.

However, because of their hydrophilic nature and low stability within the circulatory system, mimetic peptides have limited therapeutic applicability. A potential solution involves the use of synthetic polymers, which can encapsulate the mimetic peptides and enable more stable and targeted delivery to the site of action.^118^ Chen et al. showed that Cx43 mimetic peptides in poly(d,l‐lactic‐co‐glycolic acid) (PLGA) nanoparticles provided neuroprotection for up to 4 weeks, suggesting their potential for treating glaucomatous optic neuropathy.^119^

This is particularly relevant given glaucoma's link to vascular flow and chronic low‐level inflammation. Although high IOP is a major risk, repeated pressure spikes or blood pressure dips can also contribute to RGC loss and inflammation. Targeting gap junction activity with Cx43 mimetic peptides may protect both neurons and blood vessels, potentially slowing disease progression. These results suggest that targeting specific connexins could be a potential therapeutic strategy to reduce cell loss in neurodegenerative conditions.

## 7 CONCLUDING REMARKS

There is growing evidence that neuronal gap junctions are crucial for cell–cell communication in the retina. Their extensive presence in retinal circuits suggests they play several key roles in transmitting and processing visual signals. To maintain proteome homeostasis, it is essential to regulate connexin turnover through transcription, translation, and degradation. Any disruption to the connexin life cycle because of pathological stress impairs intracellular communication, leading to cellular imbalance, toxic aggregate formation, and cell death. Dysfunctional gap junctions have been linked to various neurodegenerative diseases.

The current focus is on understanding the molecular processes involved in connexin synthesis, folding, and degradation. Advances in proteomics could transform neurobiological research on gap junction communication and uncover new therapeutic targets.

## AUTHOR CONTRIBUTIONS

**Anna Pacwa**: Writing—original draft preparation; visualization. **Klaudia Mroz**: Writing—original draft preparation; visualization. **Xiaonan Liu**: Writing—review and editing; validation. **Adrian Smedowski**: Conceptualization; supervision; writing—review and editing; validation.

## CONFLICT OF INTEREST STATEMENT

AS, AP and XL were employees of GlaucoTech Co. The remaining author declares that the research was conducted in the absence of any commercial or financial relationships that could be construed as a potential conflict of interest.

## ETHICS STATEMENT

Not Applicable.

## Data Availability

No datasets were generated or analyzed during the current study.
